# Opposite aging effects among cell subsets revealed in the human transcriptome and epigenome

**DOI:** 10.17912/micropub.biology.001868

**Published:** 2025-11-11

**Authors:** Daigo Okada

**Affiliations:** 1 Stem Cell and Regenerative Medicine, Graduate School of Medicine, Gifu University, Gifu, Gifu, Japan; 2 Division of Preemptive Food Research, Preemptive Food Research Center (PFRC), Gifu University Institute for Advanced Study, Gifu University, Gifu, Gifu, Japan; 3 National Institute of Informatics, Tokyo, Tokyo, Japan

## Abstract

In our previous work, we reported a global landscape of opposite aging effects among mouse cell subsets, where each cell subset is defined as a combination of tissue and cell type, and aging leads to increased gene expression in one subset but reduced expression in another. In this study, we investigated whether opposite aging effects are also observed in human cell subsets using the database of differentially expressed genes (DEGs) and differentially accessible regions (DARs) in various human cell subsets. The results suggest that the opposite aging effects occur among human cell subsets at both the transcriptomic and epigenomic levels.

**Figure 1. The opposite aging effects among human cell subsets:**
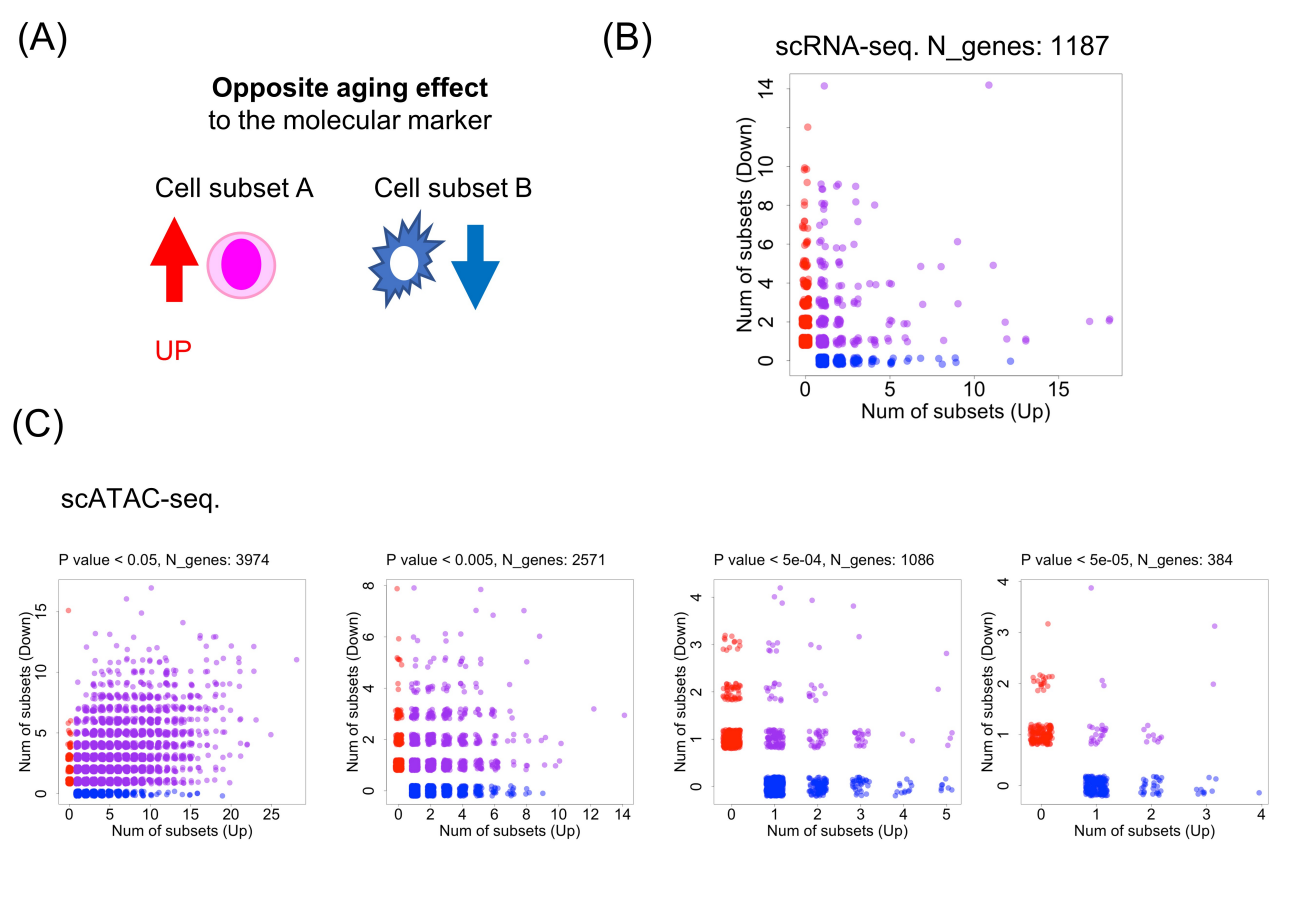
(A) Conceptual illustration of the opposite aging effect among cell subsets. (B) Analysis of age-related gene expression changes (DEGs) in scRNA-seq data. N_genes represents the number of genes included in the analysis. The panel shows the distribution of the number of subsets where expression increases with age (x-axis) versus decreases with age (y-axis) for each gene. Red dots represent Up_only genes, blue dots represent Down_only genes, and purple dots represent genes showing the opposite aging effect. (C) Analysis of age-related accessibility changes (DAR) for promoter regions in scATAC-seq data analysis under multiple P value thresholds. &nbsp;

## Description

Comparing molecular marker expression levels between young and old individuals provides a powerful framework for investigating the mechanisms of aging. Omics data analysis is particularly valuable because it comprehensively captures biomolecules that significantly increase or decrease with age. Since the human body is composed of various tissues and cell types, elucidating age-related molecular changes across diverse cell types is essential for advancing anti-aging medicine.

In our previous work using a mouse single-cell RNA-seq atlas dataset, we reported a global landscape of opposite aging effects (Okada, 2024), where aging leads to increased gene expression in one cell subset but reduced expression in another cell subset ( Fig. 1A ). Here, each cell subset is defined as a unique combination of tissue of origin and cell type, allowing us to capture tissue-specific differences in aging responses within the same cell type. In approximately 10% of mouse genes, opposite aging effects among cell subsets were observed. This finding highlights the importance of considering cell subset-specific differences when evaluating potential interventions against aging.

In this study, we investigated whether opposite aging effects are also observed in human transcriptomic and epigenomic profiles. We used the AgeAnno database, which shows aging-related markers detected across diverse human tissues and cell types using single-cell RNA-seq (scRNA-seq) and single-cell ATAC-seq (scATAC-seq) (Huang et al., 2023). The database provides lists of differentially expressed genes (DEGs) and differentially accessible regions (DARs) for multiple tissue–cell type pairs. We defined each tissue–cell type pair as a “cell subset,” since even the same cell type can exhibit distinct aging patterns depending on its tissue of origin (Kimmel et al., 2019; Okada et al., 2025). We classified the genes in the database according to the direction of age-related change.

Among all DEG records in the database, we found 1,187 DEGs between old and young individuals (adjusted P < 0.05), which covered 44 cell subsets from 5 tissues and 42 cell types. Among these genes, 18.9% exhibited opposite directions of age-related change, meaning that expression increased with age in at least one subset but decreased in another ( Fig. 1B ). These results demonstrated that the opposite aging effect exists not only in mouse but also in human transcriptomes among cell subsets.

Among all DAR records in the database, we focused on DARs between old and young individuals that were located in promoter regions (≤ 1 kb) of genes. The DARs in promoter regions were annotated to their corresponding genes. Since no adjusted P values were provided for DARs, we examined the presence of opposite genes using multiple nominal P value thresholds. The proportions of opposite genes were 78.5% (P < 0.05), 36.4% (P < 0.005), 15.9% (P < 0.0005), and 8.6% (P < 0.00005), respectively. Although constructing a rigorous statistical test is a difficult task and was not applied in this analysis, it is noteworthy that opposite genes are observed on the scatter plot even when the threshold is set strictly.

These results suggest that widespread opposite aging effects occur among human cell subsets at both the transcriptomic and epigenomic levels. A limitation of this study is that the pools of cell subsets analyzed for transcriptome and epigenome differ, making direct numerical comparisons difficult. For example, if a set containing many biologically similar cell subsets is used, the number of genes showing opposite aging effects would be smaller. Nevertheless, the key point emphasized in this study is that opposite aging effects were observed in both human transcriptome and epigenome data under standard single-cell omics analysis.

## Methods

Data analysis was performed using R version 4.3.2. The DEG and DAR lists used as source materials were downloaded on November 29, 2023, from the AgeAnno database. From the DEG table, records where the group field was “old vs youth” were extracted. From the DAR table, records where the annotation field was “Promoter (≤ 1 kb)” were selected. The procedures for calculating DEGs and DARs can be referred to in the original paper of the AgeAnno database (Huang et al., 2023).

The method for evaluating the opposite aging effects followed our previous approach (Okada, 2024). Let N_up and N_down denote the number of cell subsets in which a gene shows increased or decreased expression with aging, respectively. Genes with both N_up > 0 and N_down > 0 were classified as having the opposite aging effect. Genes with N_up > 0 and N_down = 0 were defined as UP only genes, while those with N_up = 0 and N_down > 0 were defined as DOWN only genes. We classified DEGs and DARs in the database according to these categories. When the genes exhibiting both increases and decreases were found even within the same subset, such genes were not counted as either UP or DOWN.

The analysis code is available on GitHub: https://github.com/DaigoOkada/human_opp
